# Sexual dimorphism in hepatic PPAR alpha and CYP4a12a expression is associated with reduced development of drug-induced non-alcoholic steatohepatitis in female IL-33^−/−^ mice

**DOI:** 10.3389/fmed.2024.1425528

**Published:** 2024-08-20

**Authors:** Chloe Thomas, Dolores B. Njoku

**Affiliations:** ^1^Laboratory Njoku, Department of Anesthesiology and Pain Medicine, Washington University in St. Louis, St. Louis, MO, United States; ^2^Department of Pathology and Immunology, Washington University in St. Louis, St. Louis, MO, United States

**Keywords:** NAFLD, MAFLD, ELOVL1, PPARα, lipid, metabolism, sexual dimorphism

## Abstract

Males are at higher risk for developing metabolic dysfunction-associated steatohepatitis (MASH) than females; however, mechanisms mediating sexual dimorphism in MASH development are not completely understood. Nutrition-based mouse models suggest that dysregulated fatty acid biosynthesis promotes MASH. Drugs recapitulate MASH without diet variabilities. This brief report investigates associations of sexual dimorphism with male susceptibility to MASH utilizing a drug-induced MASH model and focuses on very-long-chain fatty acid biosynthesis pathways. We assessed male and female mouse livers at 5 and 15 weeks following MASH induction by immunizations and age-matched un-immunized controls utilizing Western blot. Our results suggest that PPAR alpha and CYP4a12a protect females, while CYP4v2 does not protect males from MASH development. Our results have important implications for understanding sexual dimorphism in the pathogenesis of MASH.

## 1 Introduction

Current research suggests that obesity, abnormal circulating fats, insulin resistance, metabolic syndrome, genes, and diet may increase likelihood of developing the metabolic dysfunction-associated fatty liver disease (MAFLD). However, the precise mechanisms responsible for MAFLD are unclear. Metabolic dysfunction-associated steatohepatitis (MASH) is a severe form of MAFLD. MASH-associated liver failure has become one of the most common causes of liver transplantation in many parts of the world and is the leading cause of chronic liver disease worldwide, with a prevalence of 20%–30% in Western countries (1).

Males are at higher risk for developing MASH than females; however, mechanisms, hormonal or otherwise, mediating an exaggerated or accelerated MASH response in males are not completely understood. A major obstacle to the comprehension of MASH pathogenesis rests in phenotype variabilities in diet-induced models. Moreover, the lack of studies fundamentally evaluating sexual dimorphism as a variable has impeded the development of sex-targeted therapies. This brief report is aimed at investigating the association of sexual dimorphism of male susceptibility to MASH and focuses on pathways responsible for the development of very-long-chain fatty acids (VLCFAs).

Many mouse models of MASH are nutrition-based, and multiple mechanisms have been attributed to the disease. These models suggest that MASH results from oxidative stress and inflammation coupled with an imbalance in fatty acid biosynthesis, with or without an imbalance in fatty acid uptake and distribution. While the majority of studies have focused on fatty acid biosynthesis, pharmaceuticals (drugs) can also recapitulate critical aspects of MASH without confounding variabilities such as diet composition or variability in MASH development. Even so, drug-based models of MASH have not been fully described. In response to this need, we have developed a mouse model of drug-induced steatohepatitis in which male mice reliably develop MASH similar to male patients (2). Preliminary data in this model show strong associations of male susceptibility to MASH with alterations in fatty acid biosynthesis. Specifically, our preliminary studies utilizing targeted proteomic analysis demonstrate increased ELOVL1 in male mice that develop MASH (2), which converts long-chain fatty acids (LCFAs) to VLCFAs (3).

Although male sex has been associated with MASH, the mechanisms responsible for sexual dimorphism in MASH development have not been completely clarified. The accumulation of VLCFAs has been associated with MAFLD (4); various etiologies can contribute to this phenotype. Dysregulated lipid metabolism can promote VLCFA accumulation via overproduction from increased fatty acid metabolism or overproduction from increased LCFA conversion to VLCFAs. However, the effect of sexual dimorphism on this mechanism is poorly understood, even though male sex-associated enzymes, such as CYP4v2 (5) and CYP4a12 (6) have key roles in energy production from fatty acids and beta oxidation of LCFAs, respectively, and have been associated with MASH. Because of the vast potential of mechanisms that might have a role in sexual dimorphism in MASH development, in this brief report, we focus on dysregulated lipid metabolism via accumulation of VLCFAs that could result from overproduction from increased fatty acid metabolism or overproduction from increased conversion of LCFAs to VLCFAs. Specifically, we investigate whether sexual dimorphism in key regulators of the conversion of LCFA to VLCFA is associated with the development of MASH in male mice.

## 2 Materials and methods

### 2.1 Materials

Beta-actin mouse monoclonal antibody, M-IgG1 BP horseradish peroxidase (HRP) secondary antibody (Santa Cruz Biotechnology, Dallas, TX); blotting-grade blocker (Bio-Rad Laboratories, Hercules, CA); Bolt™ 10% Bis-Tris Plus WedgeWell™ 10-well Gels, Bolt™ 20X MES SDS Running Buffer, CYP4v2 polyclonal antibody, ELOVL1rabbit polyclonal antibody, goat anti-rabbit HRP-linked secondary antibody, PPAR-alpha rabbit polyclonal antibody, SeeBlue™ Plus2 Pre-Stained Standard (Invitrogen, Carlsbad, CA); Complete Freund’s adjuvant H37Ra (CFA) Difco Bacto (Pittsburgh, PA; GIIFNNGPTWKDIRRFSLTTL; AnaSpec, Fremont, CA); CYP4a12a polyclonal antibody, Pierce BCA Protein Assay, Ponceau S Staining Solution, Restore® Western Blot Stripping Buffer, SuperSignal™ West Pico PLUS Chemiluminescent Substrate (Thermo Scientific, Rockford, IL); pertussis toxin (List Biologicals, Campbell, CA); Tween-20 (Sigma Aldrich, St. Louis, MO); and phosphate-buffered saline, 10X Solution (Fisher Scientific, Hampton, NH).

### 2.2 Mice

Female and male IL-33^−/−^ (BALB/c background) mice were obtained from Dr. Susumu Nakae (RIKEN Center for Developmental Biology, Kobe, Japan). BALB/c mice were obtained from Jackson Laboratories (Bar Harbor, ME). All mice were 6–8 weeks old and were maintained under pathogen-free conditions at Washington University in Saint Louis School of Medicine Rodent Facility. All experiments were approved by Washington University in Saint Louis Animal Care and Use Committee, comply with the ARRIVE guidelines, and were carried out in accordance with the National Institutes of Health guide for the care and use of laboratory animals (NIH Publications No. 8023, revised 1978).

### 2.3 Induction of drug-induced steatohepatitis

BALB/c and IL-33-deficient (−/−) female and male mice were subcutaneously injected on days 0 and 7 with 100 μg of human mitochondrial CYP2E1 epitope JHDN-5 (GIIFNNGPTWKDIRRFSLTTL) covalently modified with trifluoroacetyl chloride (TFA) drug metabolites emulsified in an equal volume of complete Freund’s adjuvant H37Ra, as previously described (2). Mice were also intramuscularly injected on Day 0 with 50 ng of pertussis toxin. Mice were sacrificed at 5 or 15 weeks after the initial immunization utilizing deep sedation with ketamine and xylazine, followed by cervical dislocation. Sera and liver were removed and snap frozen for further analyses. Liver tissues were analyzed by histology, Western blot, and ELISA. Age-matched male and female unimmunized BALB/c and IL-33^−/−^ mice were utilized as controls, as previously described (2). Experiments were run in duplicate with *N* = 4 (BALB/c immunized and un-immunized; IL-33^−/−^ un-immunized) or *N* = 5 (IL-33^−/−^ female and male immunized) mice/group.

### 2.4 Histology

Liver sections (5-μm thick) were isolated from immunized and un-immunized mice, fixed in 10% neutral buffered formalin, and stained with H&E to detect macrocytic steatosis (Histoserv; Gaithersburg, MD), as previously described (2). Tissues were analyzed utilizing MASH-CRN scoring, as previously described (2).

### 2.5 Western blot

Liver tissues sacrificed at 5 and 15 weeks were homogenized in sucrose–Tris–EDTA (STE) buffer with protease inhibitor (7). Proteins were run (25 μg/lane, 172 V) using Bolt™ 10% Bis-Tris gels and SeeBlue™ Plus2 Pre-Stained Standards and then transferred onto nitrocellulose using the iBlot™ 2 Dry Blotting System. Membranes were blocked (30 min), then probed with primary antibodies (120 min, 1:500 ELOVL1, PPAR alpha, CYP4v2, or CYP4a12 polyclonal rabbit IgG), washed (PBS-Tween®-20; 0.05%), and then 1:1,000 goat anti-rabbit HRP-linked secondary antibody (90 min), washed, developed (5 min, SuperSignal™ West Pico PLUS Chemiluminescent Substrate), and then analyzed using the LI-COR Odyssey® FC Imaging System. Membranes were stripped (Restore™ Western Blot Stripping Buffer), then treated with β-actin primary antibody (1:1,000) followed by M-IgG1 BP horseradish peroxidase (1:10,000) secondary antibody. Band intensities were analyzed (ImageJ software), normalized with β-actin, and reported as primary antibody/β-actin ratios.

### 2.6 Serum analyses

Serum triglycerides (mg/dl) and free fatty acids (FFA, mM), as well as liver tissue triglycerides (ug/mg of liver tissue), were measured using high-precision, standardized biochemical assays, and a Synergy 4 Multi-mode Micro Optical Plate Reader adapted for high-throughput determination by the Nutrition Obesity Research Center.

### 2.7 Tissue ELISA

Low-density lipoprotein/very low-density lipoprotein liver tissue levels were performed utilizing cholesterol, HDL, VLDL, and ELISA kit (Abcam, Cambridge, United Kingdom). The results were standardized in μg/mg of liver tissue. All experiments were run in duplicate.

### 2.8 Statistical analysis

Multiple group comparisons were performed using a one-way ANOVA with Tukey’s *post-hoc* test. Primary outcomes, such as histology scores, were analyzed between two groups utilizing the Mann–Whitney U-test, as recommended by the statistical package (GraphPad® Prism Version 10.0.3.275 for Windows, GraphPad® Software, Inc., San Diego, CA). A *p*-value of <0.05 was considered statistically significant.

## 3 Results

### 3.1 ELOVL1 is downregulated following immunization of female mice that do not develop MASH

Sexual dimorphism in MAFLD or MASH is not completely understood. Multiple factors contribute to these diseases. Elevated VLCFAs have been associated with MAFLD following a high-fat diet (4). ELOVL 1 enables fatty acid biosynthesis and promotes fatty acid elongase activity by catalyzing the rate-limiting step in the conversion of LCFAs to VLCFAs (3). However, the role of ELOVL1 in sexual dimorphism in the development of MAFLD or MASH has not been investigated. Additionally, the role of ELOVL1 in drug-induced MASH is not understood.

**Figure 1:**
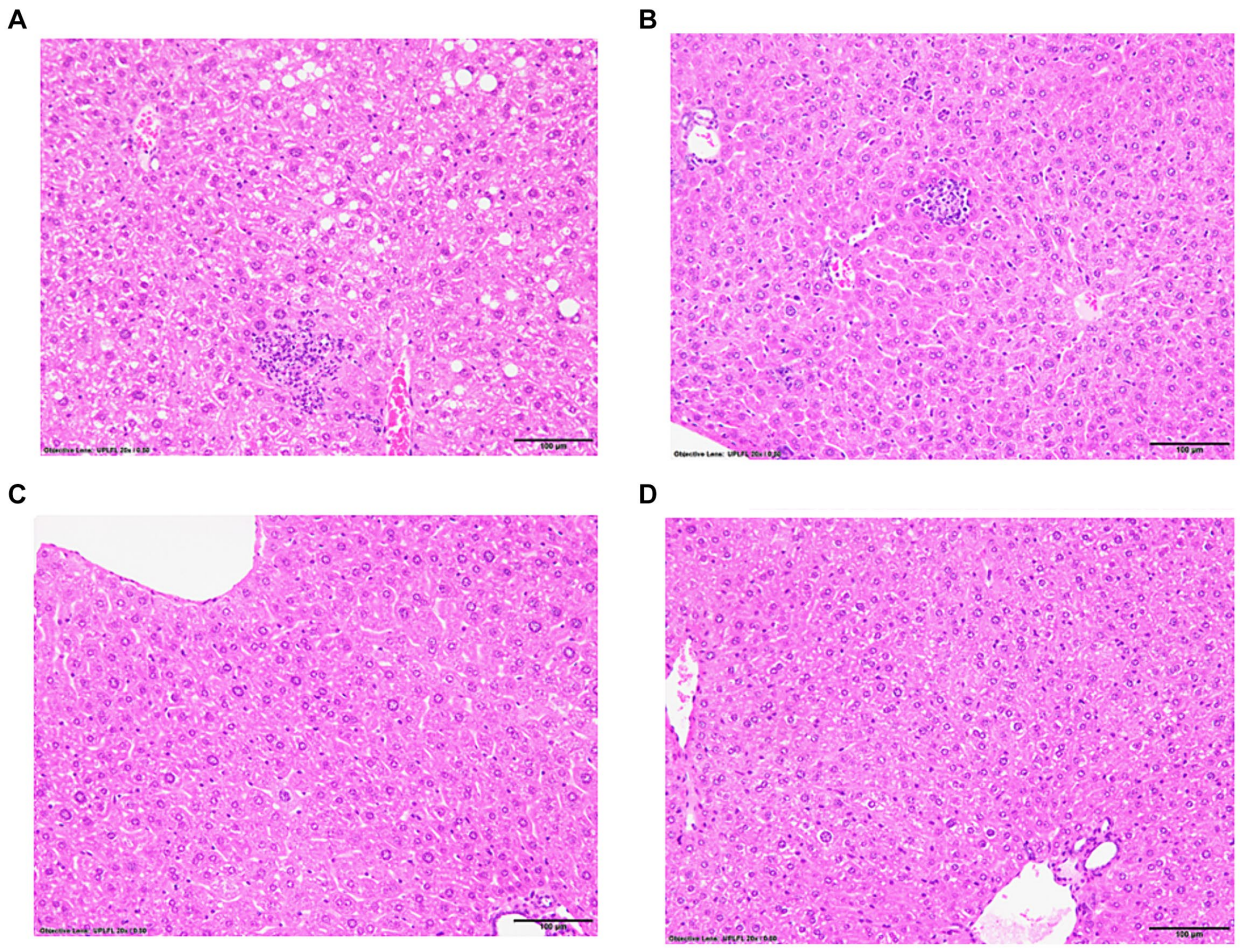
Representative hematoxylin and eosin-stained liver sections from experimental and control mice with and without immunizations to induce drug-induced MASH. IL-33-deficient (−/−) female and male mice were subcutaneously injected on Days 0 and 7 with 100 μg of human mitochondrial CYP2E1 epitope JHDN-5 (GIIFNNGPTWKDIRRFSLTTL) covalently modified with trifluoroacetyl chloride (TFA) drug metabolites emulsified in an equal volume of complete Freund’s adjuvant H37Ra and were also intramuscularly injected on Day 0 with 50 ng of pertussis toxin. Mice were sacrificed at 15 weeks after the initial immunization. Representative hematoxylin and eosin (H&E)-stained liver sections, 5 μm, demonstrating steatosis/steatohepatitis in IL-33^−/−^ immunized **(A)** males when compared to un-immunized **(B)** males. Steatosis/steatohepatitis did not develop in IL-33^−/−^ immunized **(C)** or un-immunized **(D)** female mice. 20X magnification.

**Figure 2:**
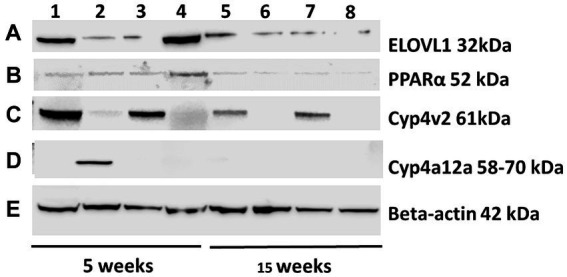
Representative Western blots were probed with ELOVL1, PPAR alpha, CYP4v2, and CYP4a12a from the experimental and control groups at 5 and 15 weeks following immunizations. Proteins from experimental and control mouse livers sacrificed at 5 and 15 weeks were separated (25 μg/lane, 172 V), transferred onto nitrocellulose membranes, blocked for 30 min, and probed with primary antibodies (1:500) of ELOVL1 **(A)**, PPAR alpha **(B)**, CYP4v2 **(C)** or CYP4a12 **(D)** polyclonal Rabbit IgG (2 h) followed by 1:1,000 goat anti-rabbit HRP-linked secondary antibody (90 min), washed, developed and analyzed. β-actin primary antibody (1:1,000) followed by M-IgG1 BP horseradish peroxidase (1:10,000) controls were applied to stripped membranes and developed. **(E)** Representative β-actin-stained membrane included. Lane 1, 5-week immunized male; Lane 2, 5-week immunized females; Lane 3, 5-week un-immunized male; Lane 4, 5-week un-immunized females; Lane 5, 15-week immunized males; Lane 6, 15-week immunized females; Lane 7, 15-week un-immunized males; and Lane 8, 15-week un-immunized females.

**Figure 3:**
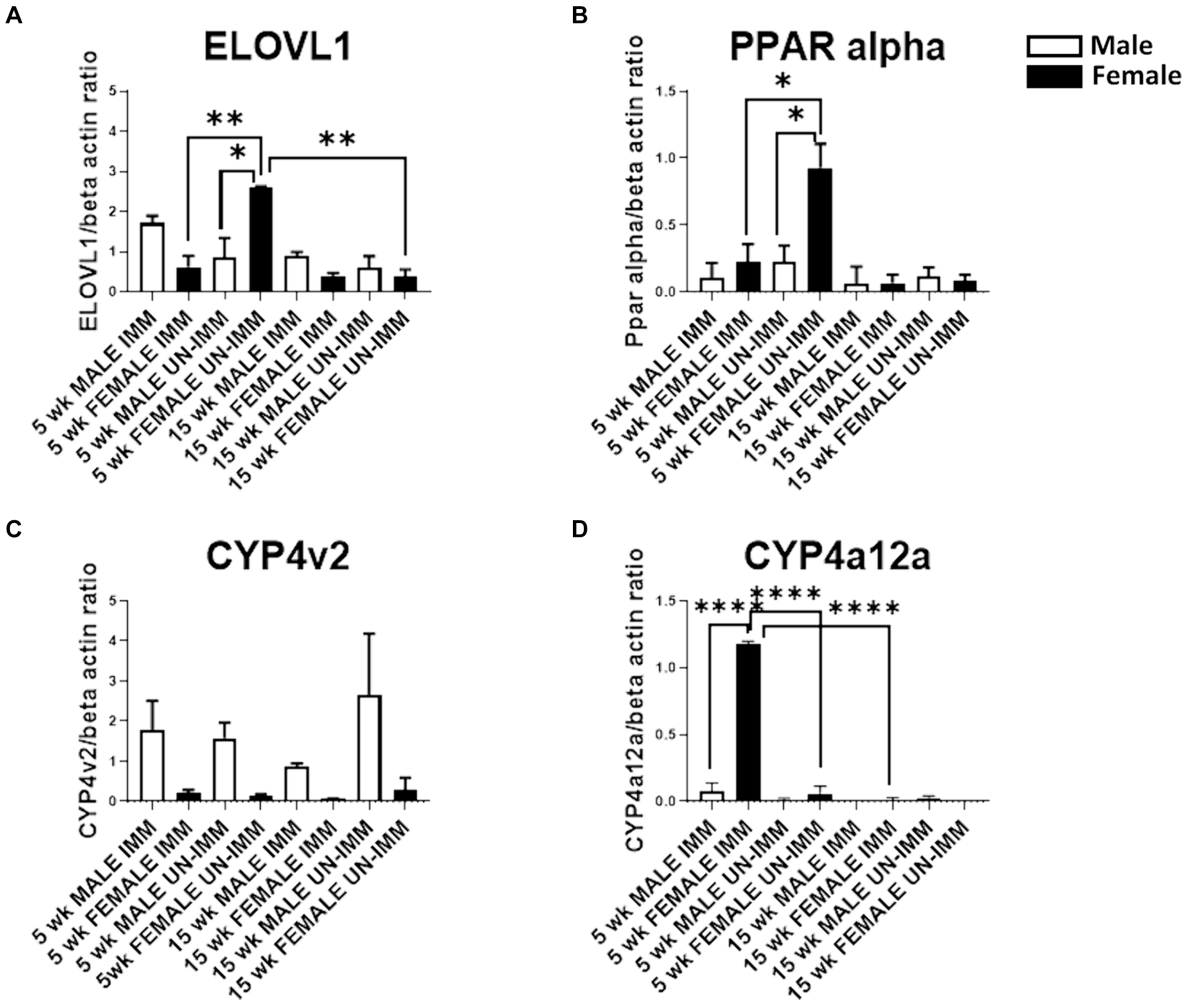
Western blot analysis of ELOVL1, PPAR alpha, CYP4V2, and CYP4a12a in livers from the experimental and control groups. Western blots were assessed as described in the methods. Band intensities were analyzed using ImageJ software, normalized with β-actin, and reported as primary antibody/β-actin ratios. **(A)** In 5-week, age-matched, female un-immunized controls, ELOVl1 was upregulated when compared to un-immunized male mice (*p* < 0.05), immunized female mice (*p* < 0.01), and un-immunized 15-week, age-matched, un-immunized female controls. **(B)** In 5-week, age-matched, female un-immunized controls, PPAR alpha was upregulated when compared to un-immunized male mice (*p* < 0.05) and immunized female mice (*p* < 0.05). PPAR alpha was similar between groups by 15 weeks. **(C)** At the 5- and 15-week timepoints, CYP4v2 expression was higher in un-immunized and immunized males when compared to un-immunized and immunized females, respectively. However, these differences did not reach statistical significance. **(D)** At the 5-week timepoint, CYP4a12a expression was upregulated in immunized female mice when compared to immunized male (*p* < 0.0001) mice, un-immunized female mice (*p* < 0.0001), and immunized female mice at the 15-week timepoint. CYP4a12a was negligibly expressed in un-immunized male mice at 5 and 15 weeks. Experiments were run in duplicate with *N* = 4 (IL-33^−/−^ male and female un-immunized) or *N* = 5 (IL-33^−/−^ female and male immunized) mice/group. One-way ANOVA, Tukey’s posttest, ^*^p < 0.05, ^**^p < 0.01, ^****^p < 0.0001.

Following immunizations, MASH developed in male (Figure 1A) but not female (Figure 1C) IL-33^−/−^ mice or un-immunized male (Figure 1B) and female (Figure 1D) IL-33^−/−^ mice, as previously described (Supplementary Figure 1) (2). We found sexual dimorphism in ELOVL1 expression at baseline, as well as 5 and 15 weeks following immunizations. Specifically, ELOVl1 was upregulated in un-immunized female mice when compared to un-immunized male mice (*p* < 0.05, Figures 2A, 3A). However, 5 weeks after immunization, ELOVL1 expression was reduced in immunized female mice when compared to un-immunized female mice (*p* < 0.01, Figures 2A, 3A). Fifteen weeks after immunizations, ELOVL1 was increased in immunized male mice when compared to immunized female mice (Figures 2A, 3A), similar to the targeted proteomic analyses that we reported in a prior study (2); however, this was not statistically significant. Interestingly, by 15 weeks, ELOVL1 expression in un-immunized female mice was significantly reduced when compared to un-immunized female mice at the earlier timepoint. These findings suggest sexual dimorphism in ELOVL1 expression at 5 but not 15 weeks in un-immunized mice. In addition, immunizations reduced ELOVL1 expression in female mice but not in male mice.

### 3.2 PPAR alpha is upregulated in un-immunized female mice

Sexual dimorphism in the regulation of fatty acid biosynthesis could modulate sexual dimorphism in MAFLD/MASH development. PPAR alpha is primarily expressed in the liver and regulates fatty acid biosynthesis via the regulation of fatty acid beta oxidation. Prior studies suggest that PPAR alpha regulates ELOVL1 (8). Similar to ELOVL1, we found sexual dimorphism in the expression of PPAR alpha. Specifically, PPAR alpha was upregulated in un-immunized female livers at 5 weeks and was diminished following immunizations (*p* < 0.05, Figures 2B, 3B). PPAR alpha was reduced in un-immunized male livers when compared to un-immunized female livers at 5 weeks (*p* < 0.05, Figures 2B, 3B). PPAR alpha was similar between groups by 15 weeks (Figures 2B, 3B). Because female mice do not develop MASH following immunizations, these findings support protective roles for PPAR alpha and suggest that sexual dimorphism in PPAR-alpha expression in female mouse livers may protect female mice from MASH development.

### 3.3 CYP4v2 does not protect male mice from MASH development

In addition to fatty acid metabolism, sexual dimorphism in oxidative respiration and/or oxidative stress could also modulate MAFLD/MASH development. CYP4v2 may promote or protect against MASH. Prior studies demonstrate that CYP4v2 promotes lipogenesis and oxidative respiration but prevents oxidative stress and the conversion of hepatic steatosis to MASH (5). We found sexual dimorphism in CYP4v2 expression in un-immunized and immunized mice. Specifically, at the 5-week timepoint, we found that CYP4v2 expression was higher in un-immunized and immunized males when compared to un-immunized and immunized females, respectively; however, these differences did not reach statistical significance (Figures 2C, 3C). Similarly, at the 15-week timepoint, CYP4v2 expression was higher in un-immunized and immunized males when compared to females, respectively, and statistical significance was not attained (Figures 2C, 3C). These findings suggested that sexual dimorphism in CYP4v2 expression was not sufficient to protect male mice from developing MASH.

### 3.4 CYP4a12a is upregulated at 5 weeks following immunizations in female mice that do not develop steatohepatitis

PPAR alpha regulates CYP4a12a, which is associated with beta oxidation of LCFA (9). Sexual dimorphism in CYP4a12 has been reported in C57Bl/6 but not in PPAR-alpha knock-out mice. We found sexual dimorphism in CYP4a12a in female mice 5 weeks following immunizations (Figures 2D, 3D). CYP4a12a expression was upregulated in immunized female mice when compared to immunized male (*p* < 0.0001, Figures 2D, 3D) and un-immunized female (*p* < 0.0001, Figures 2D, 3D) mice. CYP4a12a was reduced in immunized female mice at the 15-week timepoint when compared to immunized female mice at 5 weeks (*p* < 0.0001, Figures 2D, 3D). CYP4a12a was negligibly expressed in un-immunized male mice at 5 and 15 weeks. These findings suggest that beta-oxidation of LCFA by CYP4a12a may protect female mice from MASH development.

### 3.5 LDL/VLDLs levels are upregulated in male mice but downregulated in female mice following immunizations

**Figure 4:**
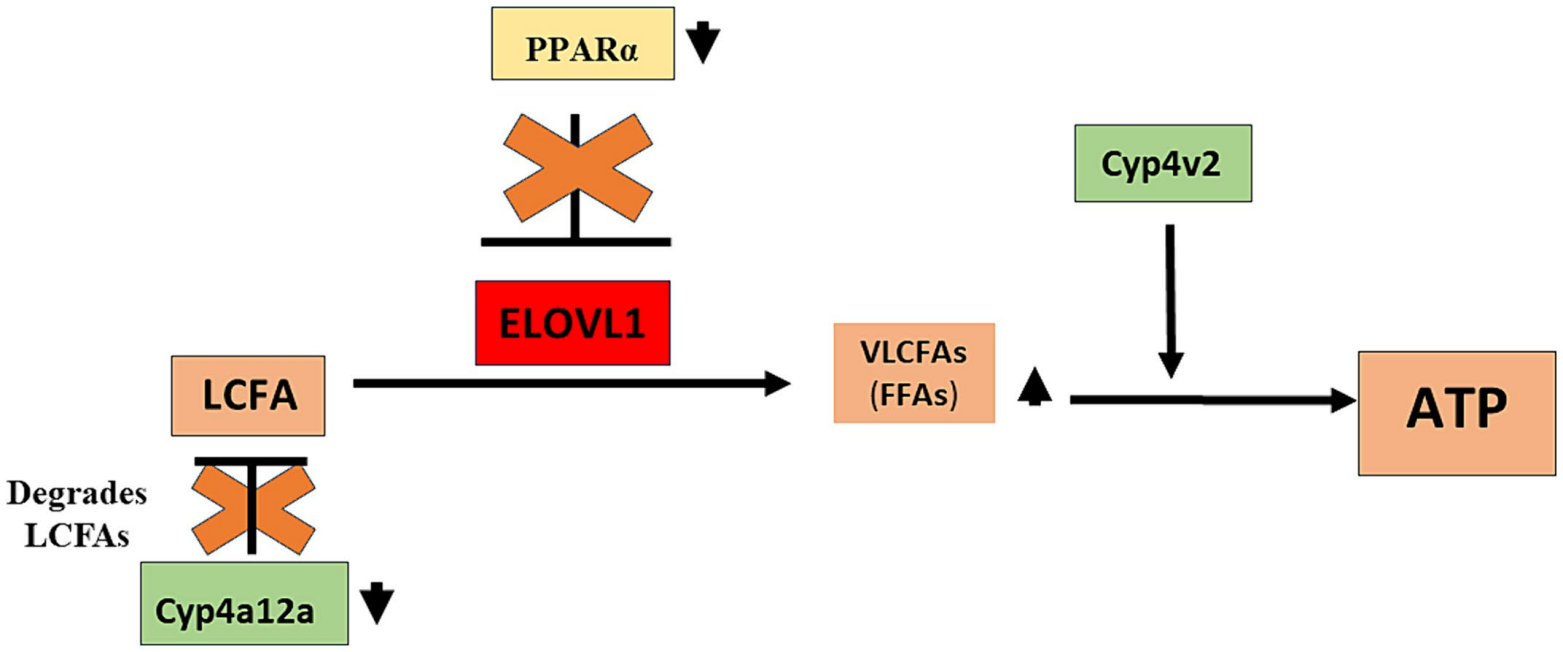
Proposed role of sexual dimorphism in the development of drug-induced MASH in male mice. Our studies demonstrate that PPAR alpha and CYP4a12a may protect female mice from MASH development and that while CYP4v2 has been suggested as protective against MASH development, upregulation of this enzyme was not sufficient to protect male mice from MASH development. We propose that in immunized male mice that develop MASH, reduced levels of CYP4a12a following immunizations provide LCFAs that can be converted to VLCFAs by ELOVL1. In addition, reduced levels of PPAR alpha in immunized male mice allow the dysregulated activity of ELOVL1 that further supports LCFA-to-VLCFA conversion. CYP4v2 protein expression although insignificantly increased without and following immunization in male mice assists in the utilization of VLCFAs for further lipogenesis and/or oxidative respiration. CYP4a12a, cytochrome P4504a 12a; CYP4v2, cytochrome P4504v2; ELOVL1, elongation of very-long-chain-fatty acids 1; LCFA, long-chain fatty acid; PPAR alpha, Peroxisome proliferator-activated receptor alpha; VLCFA, very-long-chain fatty acid.

We found that serum triglyceride levels were higher in males when compared to females in un-immunized (144.8 ± 28.0 mg/dL vs. 82.5 ± 14.5 mg/dL, mean ± standard deviation (SD), *p* < 0.05) and at the 15-week timepoint (143.3 ± 1.7 mg/dL vs. 87.1 ± 30.1 mg/dL, *p* < 0.05); however, triglyceride levels were not increased following immunizations (Supplementary Figure 2A). Serum free fatty acids (FFA) were higher in both males (2.5 ± 0.6 ug/mg vs. 0.9 ± 0.2 ug/mg, *p* < 0.01) and females (1.9 ± 0.7 ug/mg vs. 0.6 ± 0.2 ug/mg, *p* < 0.05) when compared to un-immunized mice, while levels in males vs. females following immunizations were higher but did not reach significance (Supplementary Figure 2B). Liver tissue triglyceride levels were increased in males (28.7 ± 10.7 ug/mg, *p* < 0.01) but not in females (25.8 ± 11.0 ug/mg) when compared to un-immunized males and females, respectively, and lower in un-immunized males when compared to females (17.0 ± 1.5 ug/mg vs. 24.7 ± 6.3 ug/mg, *p* < 0.01). Liver triglyceride levels were not significantly different between males and females 15 weeks following immunizations (Supplementary Figure 2C). Similar to liver triglyceride levels, liver tissue LDL/VLDL levels were lower in un-immunized males when compared to females (0.18 ± 0.05 vs. 0.36 ± 0.09, *p* < 0.01) but were increased in males by 15 weeks following immunizations when compared to un-immunized males (0.28 ± 0.05 vs. 0.18 ± 0.05, *p* < 0.05). However, in sharp contrast, LDL/VLDL liver tissue levels were reduced following immunizations in females when compared to un-immunized females (0.36 ± 0.09 vs. 0.20 ± 0.08, *p* < 0.05; Supplementary Figure 2D). Hence, although sexual dimorphism in serum and liver tissue triglyceride levels did not appear affected by immunizations, sexual dimorphism in liver tissue levels of LDL/VLDL was seen in age-matched un-immunized mice, as well as 15 weeks following immunizations, where these levels were reduced in females and increased in males following immunizations, respectively. These latter findings are aligned with our ELOVL1 and PPAR-alpha findings and strongly suggest that PPAR-alpha regulation of ELOVL1 reduces fatty acid metabolism in females via PPAR-alpha regulation of ELOVL1 (Figure 4).

## 4 Discussion

In this brief report, we demonstrate significant sexual dimorphism in ELOVL1, PPAR alpha, and CYP4a12a expression while suggesting possible roles for these proteins in the development of MASH. Specifically, our studies suggest that PPAR alpha and CYP4a12a may protect female mice from MASH development. Our studies also suggest that while CYP4v2 has been suggested as protective against MASH development, upregulation of this enzyme was not sufficient to protect male mice from MASH development. Our results have important implications for the field’s understanding of the pathogenesis of sexual dimorphism in MASH and potentially other disorders of fatty acid metabolism or chronic liver diseases.

The model described in this report utilizes IL-33^−/−^ mice, and conflicting roles for IL-33 have been demonstrated in MAFLD/MASH. A prior report demonstrated that IL-33 alleviates hepatic steatosis in high-fat diet-induced murine MAFLD (10), while another demonstrated a harmful role in the face of diabetes and MASH (11). Hence, in light of the latter study, the absence of steatosis or fibrosis in the IL-33^−/−^ mice without immunizations was surprising. Moreover, MASH development in males and not females, as demonstrated by MASH-CRN scoring, was unexpected (Supplementary Figure 1). It also cannot be ignored that it is currently not completely clear how biological sex may influence the levels of PPAR alpha, CYP4a12a, and CYP4v2, and this could be an indirect effect that simply reflects the presence of MAFLD/MASH rather than being caused by sex. Our future studies and others should determine fundamental differences in sex that will guide the development of a deeper understanding of the role of sex in MASH and possibly the development of sex-specific, targeted therapies.

MASH results from abnormalities in fatty acid metabolism leading to oxidative stress and inflammation. However, comprehensive analyses of the role of sex regarding known mechanisms of MASH in mouse models are incomplete. A landmark study by Kamada et al. demonstrated that estrogen deficiency achieved by ovariectomy in 10-week-old female C57BL/6 J mice accelerated MASH progression when combined with a high-fat, high-cholesterol diet (12). This study also described reductions in hepatic PPARα, IL-6, and macrophage inflammatory gene expression markers in these female mice. However, there was no analysis of male mice, no mention of CYP4a12a or other enzymes associated with LCFA hydroxylation, and no mention of potential mechanisms that could contribute to other extrahepatic manifestations of MASH. Our prior report of the model utilized in the current studies (2) and data support similar roles for hepatic PPARα in sexual dimorphism and protective roles for estrogen in MASH. A study by Kanno et al. (13) described roles for PPARα in MASH development in a nutrition-based model of this disease in male mice; however, sexual dimorphism or ovariectomy was not discussed. A recent model utilizing hepatic LRPPRC knock-out mice characterized by mitochondrial dysfunction in the assembly of respiratory chain complex IV and a standard chow diet demonstrated sexual dimorphism in microvesicular steatosis and cardiometabolic disease, where males demonstrated this phenotype more than females (14). However, females also demonstrated cardiometabolic impairments supported by impaired mitochondrial function and β-oxidation, as well as cardiac remodeling and elevated intracardiac triglycerides. In addition, a protective effect of estrogen was suggested without ovariectomy or androgen supplement manipulations. Thus, investigations with current rodent models are incomplete, and this deficit has deprived us of the ability to answer the most fundamental question regarding whether or not estrogen protects, and testosterone promotes MASH. Moreover, definitive evidence for PPAR alpha and CYP4a12a protection in females and the absence of protection by CYP4v2 in males would require direct interventional strategies, such as castration and oophorectomy, as well as knock-out or knock-in mice.

A recent manuscript described a comparison of A/J, BALB/c, C3H/HeJ, C57BL/6 J, CBA/CaH, DBA/2 J, FVB/N, and NOD/ShiLtJ male mice that were fed a Western-style diet enriched in fat, sucrose, fructose, and cholesterol for 8 months utilizing NASH pathology and metabolic profiling (15). They stated that a critical barrier for translation to human studies was the absence of metabolic MASH comorbidities observed in patients, such as obesity, type 2 diabetes, and dyslipidemia. Interestingly, FVB/VN mice were susceptible, while BALB/c mice were resistant to histologic steatosis, hepatocyte ballooning, inflammation, and fibrosis. Inverse relationships in lipid metabolism and chronic kidney disease were demonstrated between susceptible and resistant strains, where these parameters were increased in susceptible strains and decreased in resistant strains. Thus, we demonstrate susceptibility to MASH in IL-33-deficient male mice derived from a BALB/c background following immunization with a CYP2E1 epitope that promotes oxidative stress, inflammation, and inhibition of mitochondrial complex 1. We demonstrate dyslipidemia in mice on a resistant background strain and provide potential sex-based mechanisms that could have a role in this process. Future studies will include analyses of kidney disease and will utilize direct interventional strategies, such as castration and oophorectomy, as well as knock-out or knock-in mice in this model in addition to side-by-side comparisons with traditional NASH models in order to increase translation to human studies.

## Data Availability

The raw data supporting the conclusions of this article will be made available by the authors, without undue reservation.
