# Supplementary figures and images for: Sexual dimorphism in hepatic PPAR alpha and CYP4a12a expression is associated with reduced development of drug-induced non-alcoholic steatohepatitis in female IL-33−/− mice
Source: Front Med (Lausanne). 2024 Aug 20;11:1425528. doi: 10.3389/fmed.2024.1425528 (PMC11369704; doi:10.3389/fmed.2024.1425528)

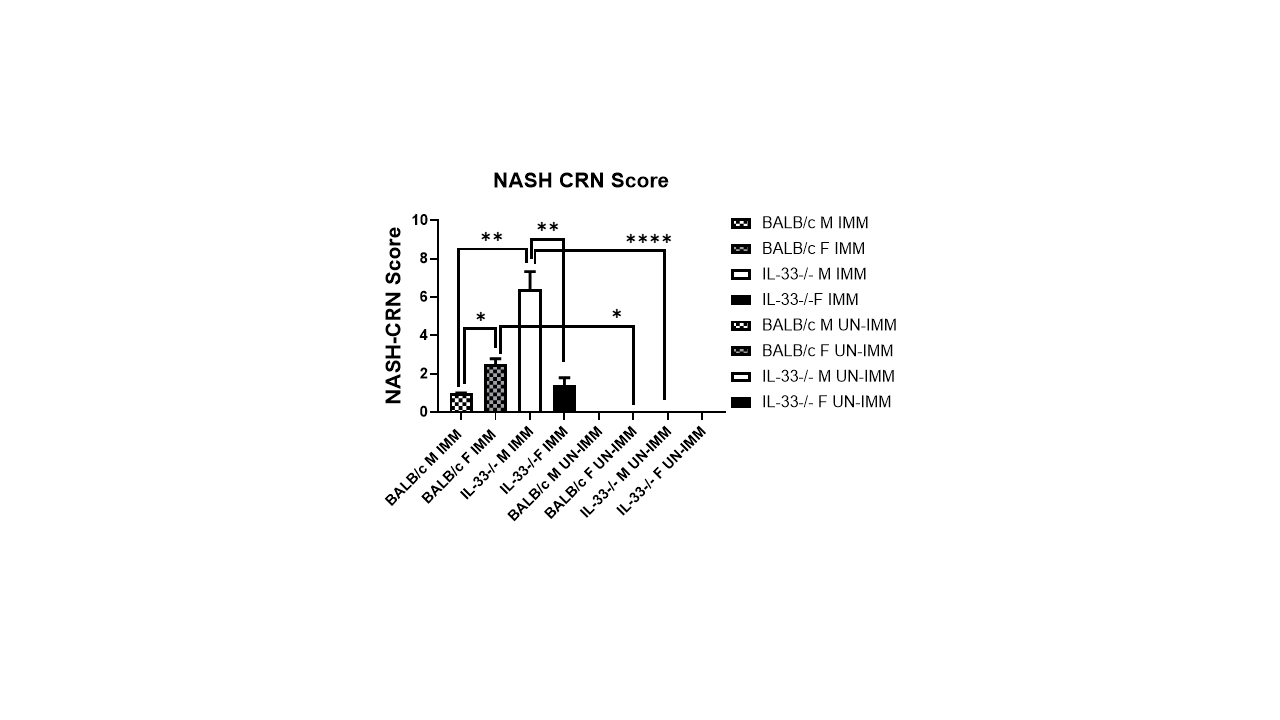

Supplement: Supplementary file 1 [file Image_1.TIF]

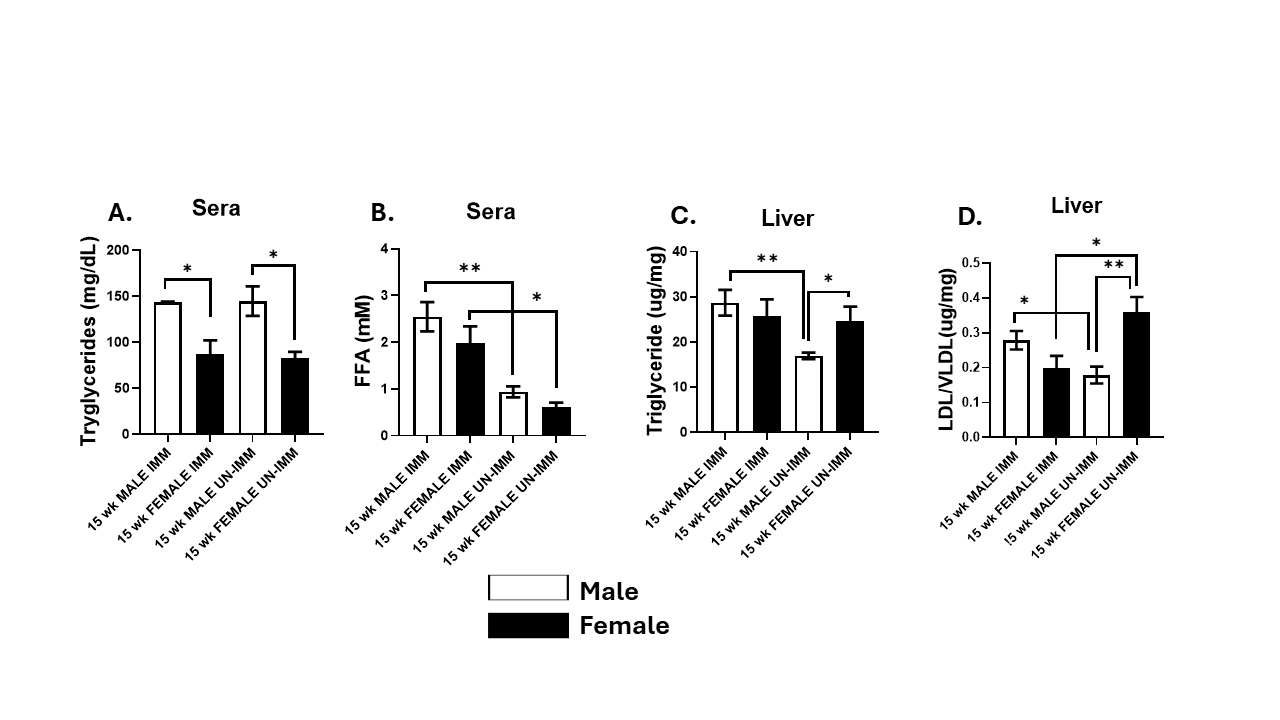

Supplement: Supplementary file 2 [file Image_2.TIF]
