# Supplementary material for: Sexual dimorphism in hepatic PPAR alpha and CYP4a12a expression is associated with reduced development of drug-induced non-alcoholic steatohepatitis in female IL-33−/− mice
Source: Front Med (Lausanne). 2024 Aug 20;11:1425528. doi: 10.3389/fmed.2024.1425528 (PMC11369704; doi:10.3389/fmed.2024.1425528)
Supplement: Supplementary file 3 [file Table_1.DOCX]

**Supplemental Figure Legends**

**Supplemental Figure 1.** Male and Female BALB/c (wild-type) and IL-33-/- (BALB/c background) mice were immunized (IMM) as described in the Methods. Un-immunized (UN-IMM) male and female BALB/c and IL-33-/- mice were utilized as controls. Briefly, BALB/c and IL-33-/- male and female mice were subcutaneously injected on Days 0 and 7 with 100 µg of human mitochondrial CYP2E1 epitope JHDN-5 covalently modified with trifluoroacetyl chloride (TFA) drug metabolites emulsified in an equal volume of complete Freund’s adjuvant H37Ra and were also intramuscularly injected on Day 0 with 50ng of pertussis toxin. Mice were sacrificed at 15 weeks after the initial immunization NASH CRN scoring was accomplished utilizing the methods of Puri and Sanyal, and demonstrated higher severity in IL-33-/- M IMM (N=5) when compared to IL-33-/- F IMM (N=5) (p<0.01), BALB/c M IMM (p<0.01) and IL-33-/- M U-IMM (N=4, p<0.0001). BALB/c F IMM (N=4) demonstrated higher severity scores than BALB/c M IMM (N=4, p<0.05) and BALB/c F U-IMM (p<0.05). Experiments were run in duplicate. One way ANOVA, Tukey’s posttest. *=p<0.05, **=p<0.01, ****=p<0.0001.

Supplemental Figure 2. Male and female IL-33-/- mice were immunized (IMM) with sera and liver tissues collected as described in the Methods. Un-imununized (UN-IMM) male and female IL-33-/- mice were used as controls. Mice were sacrificed 15 weeks after the initial immunization. **A.** Serum Triglyceride levels mg/dl were not increased by immunizations. Serum triglyceride levels were higher in males when compared to females in UN-IMM (144.8 ± 28.0 mg/dl vs. 82.5 ± 14.5 mg/dl, mean ± standard deviation (SD), p<0.05) and IMM (143.3 ± 1.7 mg/dl vs. 87.1 ± 30.1 mg/dl, p<0.05) mice. **B.** Sexual dimorphism in serum free fatty acids (FFA) were not demonstrated in UN-IMM or IMM mice or in the response to immunization. Serum free fatty acids (FFA) were higher in both IMM males (2.5 ± 0.6 ug/mg vs. 0.9 ± 0.2 ug/mg, p<0.01) and IMM females (1.9 ± 0.7 ug/mg vs. 0.6 ± 0.2 ug/mg, p<0.05) when compared to UN-IMM mice. **C.** Sexual dimorphism in tissue triglyceride levels was demonstrated in UN-IMM IL-33-/- mice where these levels lower in UN-IMM males when compared to females (17.0 ± 1.5 ug/mg vs. 24.7 ± 6.3 ug/mg, p<0.01). Sexual dimorphism in the response to immunization was demonstrated where liver tissue triglyceride levels were increased in IMM males (28.7 ± 10.7 ug/mg, p<0.01), but not in IMM females (25.8 ± 11.0 ug/mg) when compared to UN-IMM males and females, respectively. **D.** Sexual dimorphism in tissue LDL/VLDL levels was demonstrated in UN-IMM IL-33-/- mice where these levels were lower in UN-IMM males when compared to females (0.18 ± 0.05 vs. 0.36 ± 0.09, p< 0.01). Sexual dimorphism in immunization responses were demonstrated where LDL/VLDL levels were increased in males following immunizations when compared to UN-IMM males (0.28 ± 0.05 vs. 0.18 ± 0.05, p< 0.05) but reduced in females when compared to UN-IMM females (0.36 ± 0.09 vs. 0.20 ± 0.08, p< 0.05). Experiments were run in duplicate with N=4 (IL-33-/- male and female un-immunized ) or N=5 (IL-33-/- female and male immunized) mice/group. One way ANOVA, Tukey’s posttest. *=p<0.05, **=p<0.01.
